# International Stakeholder Guidance for Improving Informativeness of Randomized Clinical Trials

**DOI:** 10.1001/jamanetworkopen.2026.19487

**Published:** 2026-06-22

**Authors:** Sarah R. Prowse, Miriam Brazzelli, Heidi R. Green, Shaun Treweek

**Affiliations:** 1 Aberdeen Centre for Evaluation, University of Aberdeen, Aberdeen, United Kingdom; 2 Freelance Health Equity Specialist, Aberdeen, United Kingdom; 3 Department of Women’s and Children’s Health, Uppsala University, Uppsala, Sweden

## Abstract

**Importance:**

Many randomized clinical trials are poorly designed, address unimportant questions, or are conducted and reported in ways that limit their usefulness and contribute to research waste. Gaining insight into the perspectives of stakeholders is essential for understanding why issues affecting trial informativeness persist.

**Objective:**

To explore global perspectives on enhancing trial informativeness using a 5-point framework including trial importance, design, feasibility, integrity, and reporting.

**Design, Setting, and Participants:**

This qualitative study used semistructured, one-on-one interviews conducted via an online video conferencing platform between October 2024 and March 2025. Participants included professionals involved in the funding, design, sponsorship, and regulatory and ethical overview of randomized clinical trials. Demographic characteristics of the 55 participants were analyzed using descriptive statistics.

**Main Outcomes and Measures:**

The primary outcome was participants’ views on how the informativeness of randomized clinical trials is assessed, why uninformative trials persist, and potential approaches to reduce design and conduct flaws. Data were analyzed thematically, beginning with a deductive coding framework informed by insight from an earlier global rapid review and further refined through inductive coding to capture new themes.

**Results:**

This qualitative study included 55 individuals from 16 countries across 5 continents, primarily from the US (12 [21.8%]) and the United Kingdom (11 [20.0%]), and included investigators (21 [38.2%]), funders (12 [21.8%]), and a range of other stakeholders in randomized clinical trials. Design yielded the greatest number of insights, including further scientific design review after peer review but prior to funding commitment and the need for qualified trial statisticians. Other key insights included building trust through early and meaningful patient partnership (importance) and the need for additional skills-based training beyond good clinical practice to ensure the quality conduct of trials (integrity). Feasibility, often overlooked, was deemed an important factor, with participants stressing the need for compelling, evidence-based plans for recruitment and retention to ensure that research questions are answered reliably.

**Conclusions and Relevance:**

In this qualitative study of 55 international stakeholders in randomized clinical trials, interviews offered context and evidence to guide potential improvements in prefunding and peer review processes, as well as in trial design and best practices. Further action is needed to ensure that randomized clinical trials will consistently produce reliable evidence that improves health and strengthens public trust in science.

## Key Points

**Question:**

How can the informativeness of clinical trials be improved to ensure ethical, transparent, and meaningful research?

**Findings:**

In this qualitative study involving interviews with 55 international stakeholders, participants emphasized that trial informativeness depends on rigorous, prefunding scientific review, realistic feasibility planning, and strengthened trial integrity through training and accountability. Meaningful patient engagement and transparent registration reporting were also identified as essential to reducing research waste and enhancing trust.

**Meaning:**

These findings suggest that systematically operationalizing informativeness across the trial life cycle can ensure that clinical research produces reliable evidence that meaningfully advances patient care and policy.

## Teaser

This qualitative study uses semistructured interviews with key stakeholders involved in funding, design, sponsorship, and ethical or regulatory overview of randomized clinical trials to guide potential improvements in the prefunding and peer review processes, trial design, and best practices.

## Introduction

When researchers evaluate a health care intervention, they want to be confident that any observed changes can be attributed to the intervention itself, rather than to other confounding factors. Unlike other evaluation designs, randomized clinical trials can account for both known and unknown confounders. This feature is why randomized clinical trials are widely regarded as the gold standard for evaluating not only health care interventions but also approaches in social science and policy.

It is, however, entirely possible to conduct a poor trial, and by some accounts,^1,2^ more than half of all trials fall into this category. Existing peer review processes do not consistently identify methodologic or operational problems early in the trial, when corrective changes are still possible. Some issues, such as choosing the wrong outcomes or implementing a flawed randomization system, are virtually irrecoverable. At best, such errors result in wasted research; at worst, they risk harming participants.

In 2019, Zarin et al^3^ introduced the term *uninformative trials* to describe studies that are unable or unlikely to produce meaningful evidence. Shortcomings in trial design, conduct, and reporting can limit a study’s contribution to scientific knowledge and reduce the overall value of the resources invested.^3^ In contrast, *informative trials* meet the following 5 fundamental conditions necessary to produce high-quality evidence^3,4^:

1. Importance, defined as addressing a research question expected to inform a significant scientific, clinical, or policy decision;
2. Design, using a methodologic approach capable of producing reliable evidence to answer the study hypothesis;
3. Feasibility, defined as having a demonstrably achievable plan to enroll and retain an adequate number of participants;
4. Integrity, defined as conducting and analyzing the study according to scientifically robust principles consistent with its design; and
5. Reporting, ensuring mechanisms are in place for the timely, complete, and accurate dissemination of findings.

As part of the broader Improving Prefunding Peer Review to Increase the Informativeness of Randomized Trials (INFORM) program, which also includes a rapid review of global insights and a content analysis of trial guidance documents, we conducted a qualitative study using semistructured interviews with professionals from around the world involved in the funding, design, sponsorship, and regulatory review of randomized clinical trials.^5,6,7^ Interviews explored participants’ perspectives of trial informativeness, including how informativeness can be assessed and strengthened, guided by the following research questions: (1) How do investigators, sponsors, funders, research ethics committees, and other regulators identify and address scientific design problems? and (2) What are their perspectives on how trials containing major scientific design, conduct, and analysis flaws can be easily identified and avoided?

Gaining insight into the perspectives of those directly involved in funding, designing, sponsoring, and approving trials is essential for understanding why issues affecting trial informativeness persist. These interviews offer context and evidence to guide potential improvements in prefunding and peer review processes, as well as in trial design and best practices.

## Methods

### Study Design and Reporting Framework

We conducted a qualitative study using semistructured interviews with professionals involved in the funding, design, sponsorship and regulatory or ethical overview of randomized health care trials.^8^ Participants were all professionals in the field of trials research and contributed informed opinions within their areas of expertise. Where professional relationships already existed (eg, colleagues or collaborators), these did not involve power imbalances. At the end of the interviews, participants were offered certificates attesting to their participation in the study. A protocol was developed and is available on the Open Science Framework platform.^6^

The study received ethical approval from the University of Aberdeen School of Medicine, Medical Sciences and Nutrition Ethics Review Board; all participants provided electronic consent before being interviewed. We followed the Standards for Reporting Qualitative Research (SRQR) reporting guideline.

### Sampling Strategy and Recruitment

A purposive sampling strategy was used to achieve geographic diversity and representation across investigators, ethicists, regulators, and other relevant stakeholders. Participants were recruited globally through professional networks and connections and publicly available details on clinical trial registries and other websites. To achieve a balanced sample across continents and participant groups, we aimed for a final sample size of 50 to 70 individuals. This sample size was set pragmatically—based on practical constraints such as time, cost, or feasibility—to achieve meaningful data while keeping data collection feasible. The decision to stop would be taken when 70 interviews had been completed or when we judged that interviewees were now largely repeating points made by previous interviewees (ie, thematic saturation).

### Data Collection

Interviews were conducted remotely via Microsoft Teams (Microsoft Corporation) or Zoom (Zoom Communications Inc) at a time that was convenient for participants; each interview lasted approximately 30 minutes. A tailored topic guide, informed by the 5 conditions for trial informativeness described by Zarin et al,^3^ was used to explore participants’ views on how trial informativeness is assessed, why uninformative trials persist, and potential approaches to reduce design and conduct flaws (eAppendix in Supplement 1). Brief demographic details were also collected for all participants during the interviews.

### Data Analysis

All interviews were audiorecorded with participant consent and were transcribed using the software Quirkos, version 3, with the research team checking each transcription, and deidentified prior to analysis.^9^ Data were analyzed thematically, beginning with a deductive coding framework informed by insight from the earlier global rapid review and further refined through inductive coding to capture new themes.^5^ Coding and theme development were undertaken in full by 1 member of the study team (H.R.G.) with 2 or more team members providing quality assurance in the interpretation of results (S.R.P., M.B., and S.T.) to enhance analytic rigor and trustworthiness.

## Results

Between October 2024 and March 2025, we interviewed 55 individuals from 16 countries across 5 continents. Some participants, especially those working in the pharmaceutical industry, spoke with a global perspective rather than a country-specific perspective. One participant based in North America was part of an organization representing South America, giving us perspectives from a sixth continent. Participants were primarily from the US (12 [21.8%]) and the United Kingdom (11 [20.0%]) and included investigators (21 [38.2%]), funders (12 [21.8%]), and a range of other trial stakeholders. Table 1 illustrates not only geographical reach but also broad representation across stakeholder groups. Many investigators also contributed to multiple perspectives (eg, by serving on ethics and funding committees in addition to their primary professional roles).

**Table 1.** Participant Characteristics by Stakeholder Group and Country

Characteristic	No. (%) of participants (N = 55)
Stakeholder group	
Investigator	21 (38.2)
Funder	12 (21.8)
Ethics	6 (10.9)
Regulatory and/or standards	4 (7.3)
Industry	4 (7.3)
Contract research organization	3 (5.5)
Consultant	3 (5.5)
Operations	1 (1.8)
Sponsor	1 (1.8)
Country	
US	12 (21.8)
United Kingdom	11 (20.0)
South Africa	5 (9.1)
Australia	3 (5.5)
Canada	3 (5.5)
Ireland	3 (5.5)
Kenya	3 (5.5)
Switzerland	3 (5.5)
France	2 (3.6)
India	2 (3.6)
Norway	2 (3.6)
The Netherlands	2 (3.6)
Nigeria	1 (1.8)
Germany	1 (1.8)
Ethiopia	1 (1.8)
Italy	1 (1.8)

Illustrative quotes are presented in Table 2. Together with each quote we give the participant’s identification and self-reported professional role, country, age by decade, and sex. This makes transparent the range of perspectives represented and supports interpretation of the findings. The key themes and exemplary quotes are highlighted as relevant to actions or processes to improve trial informativeness. These themes were identified across the 5 key dimensions of trial informativeness, including importance, design, feasibility, integrity, and reporting.

**Table 2.** Key Interview Themes and Supporting Quotes as Relevant to Actions or Processes to Improve Trial Informativeness

Recommended process or action	Interview excerpts [participant self-identification]	Summary of findings
**Importance: trial hypothesis is likely to inform an important scientific, medical, or policy decision**		
Building trust through early and meaningful patient partnerships	“Involve them in the trial. Involve them in deciding what interventions are meaningful, what context and constraints should be. I think that’s key … know these people. Know their problems. Know their situations.” [Investigator, Canada, 60s, male]	Involving patients from the outset of trial design helps shape relevant hypotheses, address issues important to them, and ensure the intervention is acceptable; early collaboration fosters trust, improves feasibility, and supports stronger patient engagement in trials
	“What is now more and more done is patient involvement basically, or involvement of patient representatives, and I’m also encouraging this at the protocol stage so that at least 1 or 2 patient representatives are involved when the protocol or proposal for a funding application is drafted, so that especially the outcomes and inclusion and exclusion criteria for a trial are again checked and reviewed by patient representatives who know already some of the research processes and research methodology.” [Investigator, Germany, 50s, male]	
	“You need to build relationships with community. So, it comes up a lot in our [equity, diversity, and inclusion] initiatives that if you want people to trust health research, you need to build relationships.” [Funder, Canada, 50s, female]	
	“How do we educate the public about the importance of participating in these studies? There’s a history here of exploitation, especially of vulnerable groups of racial and ethnic minorities, and that’s I think in some ways the million-dollar question: How do we just increase the public’s willingness to participate in this sort of research? … it’s got to be multi-pronged … it’s being willing to listen and to be humbled with these groups who have been exploited and to understand why they might have aversion to research, rightly so, to build bridges and relationships with them....” [Ethics, US, 40s, male]	
Implement a formal process of research priority or agenda setting that leverages existing structures and systems	“You need to have a systematic review in place before you begin.… Is there a prioritization framework or approach in place before the scientific committee and the ethics committee even move ahead? Is there some sort of checkbox system that they can use to see if this is the priority? What does it mean anyway? Whose priority?” [Investigator, South Africa, 50s, female]	Justify trials through stakeholder consultations and evidence synthesis (including systematic reviews, rapid reviews, and other forms of evidence mapping) to develop clear research questions and ensure the topic is a recognized priority that offers real benefit to the target population
	“In the US context, I think [Europe] and especially [the] UK are far ahead of us.… Your funding application requires systematic reviews, we don’t have that requirement, we ask you for [a] literature review. And often … the trials were initiated not knowing the extent or the scope of existing literature.” [Investigator, US, 40s, female]	
Extend and formalize traditional research practices through open science	“What [a funder says] is … they have a 2-stage application plan and if you check off that you are willing to do the registered report route … you will get funding. And what makes it attractive is that if you don’t check off registered report, I think the same proportion will get to stage 2, but only half of those will get the funding.” [Investigator, Norway, 50s, male]	Preregistering prospective projects on online platforms or submitting a registered report where hypotheses, experimental design, and analytic plans are specified prior to data collection
**Design: trial methods are likely to provide meaningful evidence related to the study hypothesis**		
Conduct a scientific design review after the peer review process but prior to a funding commitment	“Sometimes if you’re industry funded, that step is not quite there, but obviously peer reviewers come in at the funding stage… To be honest, that’s a little bit of a mixed bag, because it’s very hard to put all the weight of responsibility on the peer reviewers. It’s a very crucial job, but it’s often an undervalued job, and many people are doing it at the bounds of their limits of availability. So, I think if there are major flaws, definitely peer review should pick them up.” [Investigator, UK, 50s, female]	Prior to trial funding, further consideration of the trial design and research methods are needed to enhance informativeness and reduce research waste
	“We have an additional step of review. So once the independent peer review for trials that we fund has taken place, there’s an additional, more informal review within our grant innovation platform, sort of an in-house step … they’ve also been able to identify gaps in science from time to time that have not been discovered at area levels. And we often may go back to the scientific team and ask them to address additional concerns that we have about both design and feasibility.” [Funder, South Africa, 40s, male]	
Trial teams should include dedicated expertise from a qualified trial statistician	“We have very, very few jobbing trial statisticians in Ireland, and by very, very few, when I kind of counted up how many we really have, my count is somewhere less than 10, and maybe closer to 6, people whose job, job it is to be supporting investigator-led university-sponsored trials, clinical trials.” [Investigator, Republic of Ireland, 50s, male]	Qualified trial statisticians are essential to ensure robust statistical foundations, prevent underpowered studies, and navigate complex trial designs; this expertise may be critical in low- and middle-income countries, where outsourcing may be required to access such specialized skills
	“They could be outsourcing statistical support because, for example, … I have experience [with] quite a number of teams in low- and middle-income countries, for example, in Africa, that do not have … that specific expertise, and this would be outsourced. But even when it is outsourced, they need to be competent biostatisticians as well to be able to support this.” [Funder, UK, 40s, male]	
Accountability from trial funders and/or sponsors to ensure trial design considerations are appropriate and adequately justified	“There’s guidance with the [funding] call paperwork around some aspects of things, but I would say beyond that, there’s very limited input from funders, none from the university, none from any ethics committee. I’d say that … 99.9% of the advice and responsibility for designing these studies comes from us within the study team when it’s all said and done.” [Investigator, Republic of Ireland, 50s, male]	Trial funders and/or sponsors should scrutinize the selection and validation of outcomes, interventions, comparators, sample sizes, and follow-up plans and ensure through due diligence that the trial team is prepared to implement the design effectively
	“I would stop funding underpowered trials.… We all know very well power has nothing to do with internal validity, but I just find the correlation just so strong when you’re underpowered, you have like 30 participants, it’s not really going to answer anything meaningful, and it’s likely not going to be informative, and it’s correlated with so many other features of the trial … it’s a bad trial.” [Investigator, US, 40s, female]	
Consider a diversity of expertise within trial teams to ensure conditions for informativeness are incorporated throughout all aspects of trial planning and execution	“We don’t have a trial manager or a coordinator always around, so that the trial can be conducted smoothly and someone is really taking responsibility, so a lot of trials here don’t work out and do not produce useful results because of these little organizational problems …” [Investigator, Germany, 50s, male]	Informative trials rely on a coordinated team including trial management, community perspectives, data analysis and other operational expertise, working collaboratively to maximize impact and deliver results efficiently
Implement staged funding that supports trial design and delivery	“We never disperse the full amounts at the beginning. We give funding for people to commence the work, usually up to 50% of the funding in the beginning and then the rest of the tranches are based on certain milestones being achieved.” [Funder, South Africa, 40s, male]	Resources are released in phases based on trial progress, as assessed by the appropriate stakeholders
	“Funders could put in funding milestones and say that we’ll give you the funding to design the trial, but further funding is dependent upon that having satisfactory review, not just ethics committee review, but also for informativeness.” [Investigator, US, 70s, male]	
Select fit-for-purpose tools to further support a process of informed trial design	“I’ll use tools that I know I will need for publication, and I also will ask my team to use similar tools, so when they’re thinking about collating data around screening numbers and et cetera, then I would then say okay, our end goal is the CONSORT table et cetera, I’ll have in mind different checklists that I need to get through in order to design a study through the EQUATOR Network et cetera, depending on the type of trial that it is, and I will confer with colleagues where I don’t have the expertise in terms of more innovative trial designs or partner with people who are more used to doing those kind of trials, to increase, to ensure that I’m actually adopting the right methodology when I’m using a new type of trial.” [Investigator, Kenya, 40s, female]	Trial designs can be further improved through the use of appropriate design tools and frameworks or other guiding documents intended to reduce research waste
Establish research forums and other collaborative networks as a source of trial design feedback and support	“I would also include right at the start as many of the appropriate stakeholders as possible, so who am I trying to inform.… I want them in at the start because I need to understand how they’re going to use that data, so I would, if possible, get patients and public, definitely speak to clinical colleagues and generally speak to health service managers and policymakers because … I as an investigator want anything that comes out of my trial to be instantly usable, so I think it’s really important to have that right up front.” [Investigator, UK, 50s, female]	Research forums and collaborative networks can strengthen trial design and foster productive relationships with funders; building these networks early supports funding success and facilitates shared learning
If appropriate, consider pragmatic trial design approaches that evaluate the effectiveness of interventions under routine practice conditions	“Instead of making clinical trials more complex and difficult to deliver, making them more pragmatic, depending on the level of product that we are dealing with, the type of disease that we are dealing with.” [Investigator, Ethiopia, 40s, male]	Careful consideration of trial design ensures its appropriateness, including the degree of pragmatism, in relation to the study objectives and the intended application of results
	“I would be strongly supportive of aligning the design with the intention; I’d be strongly in opposition to the idea that everybody should be only doing pragmatic trials. That is the alignment between what the purpose of the trial is and how you design the trial, and any of these agents may well be wanting to say for themselves what the purpose of the trials should be.” [Investigator, Canada, 60s, male]	
Establishing a connected ecosystem of trial funders	“I think what would be of value would be a really consistent approach across funders as to what an informative trial should be. Because the more we are collectively consistent, the more that kind of sets the standards for how trials are developed.” [Funder, Canada, 50s, female]	Connecting funders aligns standards and facilitates shared learning, improving the informativeness of trials
Incorporate future-proofing measures into trial designs to account for rapid technological advancements	“I think the external environment is advancing at such a crazy pace at the moment. If the funding application actually kept pace with that, how those technological changes impact your research question might be critical, given the exponential rate at which technology is evolving. What is the impact on what you’re doing? Are you using technologies in your trial that might be outdated by the time you answer the question, or are you using technologies that are likely to be biased instead of doing something manually, considering how that external landscape is evolving? How you will keep pace with it?” [Industry, UK, 50s, female]	Ensure trial technologies remain relevant, reliable, and free from emerging biases despite the fast pace of technological change
**Feasibility: the trial must be demonstrably feasible (eg, it must have a realistic plan for recruiting sufficient participants)**		
If appropriate, consider a pilot or feasibility study to avoid research waste and derisk funding investments	“The feasibility pilot would be part of a large piece of work, but we do definitely fund them [and] see the value in them to ensure that the methods are actually feasible, because I think a lot of the time things look really great on paper and try and do it in real life and they just kind of fall down … or you try and translate from a high-income context to a low-income country context and the methods just don’t quite translate.” [Funder, UK, 40s, female]	Feasibility studies were found to be potentially useful in assessing whether a more expensive, large-scale trial is merited
	“We’ve chosen a design in the past that hasn’t worked, that hasn’t tested the feasibility of recruitment … and it wasn’t until we started we realized there wasn’t equipoise between the [clinicians] and they wouldn’t randomize. We [learned] from that, and we then went back and really consulted widely and we changed the design, and we set up a new trial with a [new design] which is designed to help the participants, the [clinicians] … we’re now at [long-term] follow-up. So, we [learned] the hard way that sometimes … if you don’t do enough feasibility work, you will not get the result that you want.” [Investigator, UK, 50s, female]	
	“We tend to be optimistic people, but sometimes we have to question that to make sure that [the trial] is really doable.… Often we just do things because they are feasible, not necessarily because they’re the most informative.” [Investigator, France, 40s, male]	
	“Because in most of the [cases] … the scientific question is a good one. But then how the trial will be implemented, whether it was checked that maybe the investigator site [has] the right patient, how this will be done, whether this will be accepted in the country or not, this is missing. And a few of the people, I wouldn’t say everybody, but I think the people who are most associated with operations, flag operations as an area where academics perhaps in particular fall down.” [Industry, France, 60s, female]	
Include community members in participant recruitment and retention strategies	“I want to emphasize the role of community advisory boards in trials and really say that they should truly become component parts of [the] design of many of our trials.” [Investigator, Nigeria, 40s, male]	Greater use of community involvement, such as advisory boards and patient and public input, is needed to strengthen recruitment and retention by improving communication and supporting sustainable trial enrolment
	“I think it’s the feasibility question. If I had to pick one [thing to improve], it would be really honing down on asking applicants to provide a compelling case for how they will recruit, target, and retain the participants, because without that it doesn’t matter how valid the methods are, how great the question is, nothing informative will come of that.” [Investigator, Canada, 40s, male]	
	“The achievement of the target number and patient and public involvement are probably key to whether we can actually achieve [the trial] or not.” [Sponsor, Scotland, 60s, female]	
Utilize the knowledge of local health professionals to better inform feasibility or pilot studies	“Have they actually engaged with any, if it’s in health care, any health care services to see if they’re you know willing to refer people to the study? Things like that, but I think perhaps we don’t ask but should be done and check the feasibility before applying.” [Funder, UK, 40s, female]	Integration of local health professionals within the trial process can further contextualize issues of recruitment and retention and help to ensure that the required sample size can be reached
Integrate qualitative evidence when assessing the feasibility of a trial	“Considering all the problems you have with patient involvement … the research group can plan to do … extensive qualitative work. But I think that’s [the] rare case…. Sometimes they gather … the interest groups or the stakeholders, whatever … and you have … a series of 5 [group] discussions, whatever. And you know … that’s good, that’s cool, that can be better.” [Investigator, Norway, 50s, male]	Planning for and integrating qualitative evidence can enhance trial feasibility and is an approach that merits further consideration and adoption
**Integrity: the trial must be conducted and analyzed in a scientifically valid manner that is faithful to the design**		
Further development of skills-based training to ensure the quality conduct of trials	“I’m interested in the training aspect of it and what training people get around trials. Apart from their experience of doing them, they’re kind of on-the-job training and people recognizing those aspects of informativeness and why they’re important.” [Funder, Republic of Ireland, 50s, female]	Trial teams may benefit from training that extends beyond standard good practice guidelines, with continuous, structured learning planned from the outset to build the specific competencies required for high-quality trial conduct
	“The simple lack of training that clinicians get in doing randomized trials is leading us to endlessly [produce] the same kind of trial that we have done in the past, which is a trial that isn’t asking clearly what the purpose of the research question is, it’s not asking what this trial is a tool, what is this tool trying to achieve.” [Investigator, Canada, 60s, male]	
Ensure effective trial oversight	“After funding, that’s when your trial steering committee I think becomes a very useful oversight body and also your data monitoring committee, they are crucial in identifying if anything is going awry and also providing that input to whether a remedial action should be taken on board or not.” [Investigator, UK, 50s, female]	Undertake appropriate ethics review, maintain active governance through a trial steering committee, and uphold context-appropriate data protection to ensure trial compliance and integrity
	“I think that there should be robust scientific review, and [that should occur] before it comes to the ethics committee. And nevertheless, the ethics Committee should be able to question the science further. So, if they continue to have questions, that is their right.” [Investigator, US, female]	
Adequate facilities, resources, and staff capacity to deliver and sustain trials	“One thing that we we’ve been thinking a lot about is clinical trial readiness for clinical sites. So, getting the sites themselves set up as well as they can be, so you know you have all of those different types of expertise that are needed.” [Funder, US, 45, female]	Trial sites should have the physical infrastructure, equipment, and skilled personnel needed to deliver the protocol; this includes building capacity among current and future staff to sustain trial operations
	“Quite frankly, I would need to dwell more on the building capacity for trials, and I would say so especially in the context of the parts of the world where I live where I strongly feel that we don’t have … sufficient programs that [train] people in conduct of [trials].” [Investigator, Nigeria, 40s, male]	
Registration of clinical trial units within wider national or international networks	“Several stakeholders could take responsibility here and probably clinical trial units have a crucial role so that when people discuss research questions, or already the outline of a trial, that it becomes clear that there should be elements like how can the results actually be applied?” [Investigator, Germany, 50s, male]	Safeguards capacity for the development and delivery of high-quality trials by ensuring key competencies for conduct are met within a clinical trial unit under the guidance of appropriate expertise
**Reporting: systems are in place to ensure timely, complete, and accurate reporting**		
Dissemination of trial outputs and wider knowledge mobilization	“Reporting to the participants … I can’t imagine, if I were a trial participant not knowing the results, having to hunt for the publication, that would drive me [completely] bonkers.” [Investigator, France, 40s, male]	Trial findings should be shared through accessible, inclusive formats with relevant audiences as appropriate including the trial participants; broader knowledge mobilization should promote the uptake of results into policy and practice at local, national, and/or international levels
	“How will it be implemented? Or conversely, if treatment A is not necessarily shown to be better than B, what is the implication for practice and future research?” [Investigator, US, 40s, male]	
Mandatory requirement to proactively register trials alongside a supporting system of monitoring to ensure compliance with registration, up-to-date record keeping, and timely publication of results	“Speaking about the reporting and making sure that actually the results, like all results, are reported, not just those that are positive because I think often if the trial is negative, they don’t get reported and that can really impact on the field.” [Funder, UK, 40s, female]	Publicly specifying details on trial methodology and conduct before enrolling participants increases transparency, decreases selective reporting and subsequent publication bias, and ensures an ethical responsibility to publicly report trial results
	“Our main problem still today is publication and reporting biases, so it’s changes of switching outcomes without telling about it, it’s selective reporting on just parts of your population, it’s not reporting your results at all, so I would say that making the registered report format something that you always have to do when you’re doing hypothesis testing and drawing statistical inferences, that should be the main change.” [Ethics, Norway, 40s, male]	
	“Some other aspect that I think is important … is around registration of the clinical trials in public registries. … because that provides a platform for the scientific community and the public as well to be able to understand the context of these clinical trials and how they can be able to contribute to the different objectives that they’re trying to address.” [Funder, UK, 40s, male]	
Reporting of health equity considerations	“I’m also a massive advocate for [equity, diversity, and inclusion] … there’s not one national framework.… Without that and some sort of mandate to do it, it’s difficult.” [Funder, UK, 40s, female]	Transparency and completeness within the trial reporting process should consider how the presentation of the intervention and trial results may influence policymaking and other decisions for those who are currently underserved by health research and health services

Abbreviation: UK, United Kingdom.

Design yielded the greatest number of insights, with participants highlighting gaps in current processes. This included further scientific design review after peer review but before funding commitment as a distinct and more focused stage of assessment complementing standard peer review processes and the need for qualified trial statisticians. Beyond design, participants highlighted early and meaningful patient partnership as central to building trust, a key theme within importance, alongside the need for additional skills-based training beyond good clinical practice training to support high-quality trial conduct as part of integrity. Feasibility-related insights focused on challenges in recruitment, retention, and the practical delivery of trials, while reporting themes emphasized prospective registration and the comprehensive dissemination of trial results. When asked whether there was one thing that could be done to improve trial informativeness, 24 interviewees (43.6%) focused on aspects of design, again emphasizing the need for scientific design review after peer review but prior to funding commitment, the importance of diversity within trial teams, and accountability from those who fund trials to ensure design considerations are appropriate and adequately justified.

## Discussion

This qualitative study highlighted stakeholder perspectives on the processes and actions considered important to trial informativeness. The strongest emphasis was placed on design, alongside contributions related to importance, feasibility, integrity, and reporting.

The views expressed by interviewees were broadly consistent with a previously published rapid review of global insights on trial informativeness that included members of our team.^5^ In particular, the emphasis on research prioritization, transparent trial registration and reporting, and clear justification of key design choices reflects findings across the global literature and aligns with internationally published clinical trial guidance documents from funders, regulators, and ethics bodies.^7^ While participants’ perspectives acknowledged recognized standards, they also offered more nuanced insights into where improvements are needed and how trial informativeness can be further operationalized in practice. This furthers earlier work^5,7^ by providing stakeholder perspectives on how key aspects of trial informativeness are interpreted and applied in practice, while also highlighting areas where improvements are needed.

Clear differences emerged between how industry and academia approach trial importance, reflecting their differing objectives. For industry, the emphasis was typically on informing drug pipelines or assessing safety or efficacy, whereas academia was more focused on contributing to complex discussions aimed at shaping policy. Both groups, however, recognized the need for a formal process of research prioritization or agenda setting to ensure that research questions are clear and well-justified. Examples of mechanisms to support prioritization included stakeholder consultations and evidence syntheses, including systematic reviews, rapid reviews, and other forms of evidence mapping. Engagement through meaningful patient partnerships was the most widely recognized aspect of importance across the interviews, including navigating the complex challenges of historical mistrust that often accompany participation in trial research, particularly for underserved groups.^10^

In considering the design aspect of informativeness, interviewees agreed on the need to conduct a scientific design review after the peer review process, but prior to a funding commitment to reduce research waste. There were differences between industry and other stakeholders, with the former more likely to implement this practice to safeguard large financial investments and ensure designs meet regulatory and commercial requirements. Industry representatives also discussed the use of pretrial modeling, including simulations and predictive analyses to inform judgements about the value of doing a trial, given plausible treatment effects, to optimize sample size and timelines, and to inform risk-benefit considerations before a trial begins.^11,12,13,14^ The interviews also highlighted the clear need for trial teams to include dedicated expertise from a qualified statistician, a finding echoed in wider research.^15,16^ However, as described in Table 2, this is not always easy, given a lack of suitably qualified candidates.

A key implication of these findings is that feasibility remains underemphasized in funding and design processes, despite its central role in determining whether trials can generate meaningful results. A lack of feasibility due diligence, coupled with a weak justification in trial proposals for why specific sites, recruitment, and retention targets and timelines are feasible, was identified as an important reason for poorly informative trials. It was unclear why feasibility is often neglected. One explanation may be that funders do not explicitly require it, and those who write grant applications are rarely the same individuals who know most about trial operations. At present, securing grants is largely about making a scientific case, rather than demonstrating the practicalities of delivery and operations. If trials are to become more routinely informative, this balance needs to be reassessed. A further concern was that conservative, status quo choices may lead to feasible but poorly informative trials. Delivery, therefore, must be feasible and explicitly tied to requirements that come from importance and design.

Views on integrity reflected a need for the development of additional skills-based training beyond standard good clinical practice. Interviewees emphasized the value of continuous, structured learning planned from the outset to build the specific competencies required for high-quality trial conduct. Insights also highlighted the trial team as a wider whole, including those responsible for on-site patient care, such as research nurses and clinical research coordinators.

There has been growing recognition of tailored training for investigators whose primary background is clinical practice, a sentiment also shared by some interviewees.^17,18,19^ The appointment of investigators with the necessary training and experience is critical both for safeguarding participants and for maintaining the quality and integrity of trial data.^17^ This point is also connected with a key aspect of design, where participants described the role of funder accountability and due diligence to ensure the trial teams are adequately prepared and equipped with the skills required to implement the design effectively.

Although reporting was less discussed than other aspects of informativeness, interviewees nonetheless acknowledged the importance of proactively registering trials and the ethical responsibility to publicly report trial results, alongside broader knowledge mobilization that promotes the uptake of results into policy and practice. Publishing trials irrespective of outcomes reduces research waste, prevents duplication, and strengthens the evidence base by mitigating publication bias.^20,21,22^ Reporting transparency ensures that all trial outcomes contribute to scientific progress and support more informed decision-making in clinical practice and research.

Because trial informativeness remains an evolving concept, these findings provide stakeholder-informed insights into how its key dimensions are understood and applied in practice. This work is being advanced through the INFORM program, including a mapping exercise integrating findings across research strands (literature review, content analysis, and stakeholder perspectives) and the development of freely available training and dissemination materials. Further efforts will focus particularly on funders as the key stakeholder group. Since funding committees play a central role in prioritizing research for funding and assessing study quality, making these processes easier and more effective is likely to increase the number of informative trials.^23^

Our interviewees collectively offered concrete suggestions for where change can be made across the 5 domains of informativeness described by Zarin et al.^3^ To focus on just 5 priorities, we believe that everyone involved in randomized clinical trials should pay closer attention to (1) trial rationale (ie, Why this trial, and why now?); (2) study population (ie, How do we know that the trial is targeting the right participants?); (3) feasibility (ie, How do we know this team can deliver this trial successfully?); (4) statistics and analysis (ie, Who within the team has the statistical expertise to design and analyze the trial appropriately?); and (5) transparency (ie, How do we know the results will be made publicly available?). Greater attention to these issues would help improve trial informativeness, and the next stage of the INFORM project will focus on how these actions can be implemented in practice.

### Limitations

This study has some limitations. Semistructured interviewing offers the benefit of generating rich, in-depth insights that capture the perspectives and experiences of participants while allowing flexibility to explore additional themes. Although participants were drawn from diverse geographies, we did not aim for an equal distribution of professional experience or other characteristics such as sex and ethnicity. Moreover, as we invited participants to self-report their country, age, sex, and ethnicity, it is likely that some people may have interpreted these questions differently. Comparison across these characteristics is therefore not straightforward. However, insights were broadly consistent across groups, with only marginal variances noted among those with industry experience. Some interviewees from lower-resource settings noted a lack of infrastructure as influential to trial informativeness, yet overall responses were broadly uniform, reflecting common needs and experiences irrespective of global setting.

## Conclusions

In this qualitative study of 55 international trial stakeholders, enhancing the informativeness of clinical trials was viewed by interviewees as not only a scientific imperative but also an ethical one, ensuring that the resources invested and the trust placed by participants translated into knowledge that meaningfully advanced patient care and policy. Although there were some differences between industry and academic perspectives, there was broad consensus that trials must be designed, delivered, and reported with greater transparency and accountability to ensure they always address important unanswered questions to reduce research waste.

All individuals who work on randomized clinical trials—investigators, funders, approval agencies, regulators, journal editors, and others—have a role to play in redesigning (or, where necessary, stopping) trials that are poorly justified, poorly designed, unlikely to be feasible, lacking adequate statistical expertise, or insufficiently transparent about how their results will be made public. As our interviewees made clear, uninformative trials are a global problem. Further action is needed to ensure that randomized clinical trials will consistently produce reliable evidence that improves health and strengthens public trust in science.

## Notes

Supplement 1. **eAppendix.** Improving the Prefunding and Peer Review Process to Increase the Informativeness of Randomized Trials (INFORM) Topic Guide and Reference for Interviewer

Supplement 2.
Data Sharing Statement
