# Supplementary material for: International Stakeholder Guidance for Improving Informativeness of Randomized Clinical Trials
Source: JAMA Netw Open. 2026 Jun 22;9(6):e2619487. doi: 10.1001/jamanetworkopen.2026.19487 (PMC13288747; doi:10.1001/jamanetworkopen.2026.19487)
Supplement: Supplement 2. — Data Sharing Statement [file jamanetwopen-e2619487-s002.pdf]

Data Sharing Statement

Prowse. International Stakeholder Guidance for Improving Informativeness of Randomized Clinical Trials. *JAMA Netw Open*. Published June 22, 2026.
doi:10.1001/jamanetworkopen.2026.19487

Data
Data available: No

Additional Information
Explanation for why data not available: Ethical restrictions prevent sharing of the underlying qualitative interview data; anonymized excerpts are included in the manuscript.
