# Supplementary material for: International Stakeholder Guidance for Improving Informativeness of Randomized Clinical Trials
Source: JAMA Netw Open. 2026 Jun 22;9(6):e2619487. doi: 10.1001/jamanetworkopen.2026.19487 (PMC13288747; doi:10.1001/jamanetworkopen.2026.19487)
Supplement: Supplement 1. — eAppendix. Improving the Prefunding and Peer Review Process to Increase the Informativeness of Randomized Trials (INFORM) Topic Guide and Reference for Interviewer [file jamanetwopen-e2619487-s001.pdf]

Supplementary Online Content

Prowse SR, Brazzelli M, Green HR, Treweek S. International stakeholder guidance for improving informativeness of randomized clinical trials. *JAMA Netw Open.* 2026;9(6):e2619487. doi:10.1001/jamanetworkopen.2026.19487

This supplementary material has been provided by the authors to give readers additional information about their work.

eAppendix. Improving the Prefunding and Peer Review Process to Increase the Informativeness of Randomized Trials (INFORM) Topic Guide and Reference for Interviewer

© 2026 Prowse SR et al. *JAMA Network Open*.

eAppendix. Improving the Prefunding And Peer Review Process to Increase the Informativeness of Randomized Trials (INFORM) Topic Guide and Reference for Interviewer

Topic Guide

Brief introduction
Firstly, thank you for agreeing to take part in this interview study and for your time today. In our study we are looking at what makes a trial useful (sometimes called informative) and how the number of informative trials can be increased. For the avoidance of doubt, we are not interested in explicit design choices or research misconduct in this study. We are also most interested in what can be done to make a trial informative *before* it is funded.

I want to emphasize that there are no right or wrong answers; we really want to hear your views. You will not be identifiable in any outputs, and we will be in touch if we would like to include anything you say today as a quote in any publication, anonymized. Do you have any questions before we begin?
Remember that although having the camera on is helpful, you are free to switch it off.

Consent
{if participant has not completed econsent:}
Firstly, I will take you through a formal consent process, I need to audio record this – is this OK with you?

I am going to read some points to you and ask you to say yes or no to each of the points. I will record your answers and initial each of the points on your behalf. Once we have finished, I will give you a completed copy of this form for you to keep.

If you have any questions at any point, please just ask.

{consent form has been emailed to participant ahead of interview in case of technical issues so either refer to it or screenshare before proceeding}

{Switch on audio recorder if OK with participant}

Please confirm your name for the recording [Participant name]
I'll add that your ID number is [relevant ID number]
{Go through the consent form}

{Stop recording of consent}
{Start recoding interview – state participant ID no. again for the recording}

Q1 In what way do you work with clinical trials?

{Confirm with all participants what their role is before proceeding with the interview}

If participant is an investigator:

Q2 Starting with a general question, what do you think makes a trial useful?

{if the respondent doesn't mention trial users (e.g., health professionals, patients) in the response, ask:

-Who are you thinking of when talking about a trial being useful? Who is the person(s) finding it useful?}

- Some US researchers created the term 'informativeness' to describe a trial that produces useful results for its users. Informativeness has five components: importance – design – integrity – feasibility – reporting. These are the things we think of when talking about the informativeness of trials.

{be ready to explain these five components if needed}

© 2026 Prowse SR et al. *JAMA Network Open*.

Q3 As an investigator, what sort of things do you do to make sure your trial is informative?

{Use the following probe if the participant struggles or does not talk about these:
--Do you do anything to avoid design problems in your trials? (e.g. use tools)

is identified).

-In your experience, at what point in the lifespan of a trial have issues come to your attention- pre-funding, funding, conducting, reporting stages?}

Q7 What do you think would be the most important change that can be made to increase the number of useful/informative trials?

Q4 How would you like to learn about what you can do to improve the informativeness/usefulness of your trials?
(for example: feedback on individual trials/in person training/online - videos/quizzes/accredited CPD courses)

Q5 Who/which organizations do you think are best placed for making sure a trial design is as good as it can be?

Q6 Are there organizations that you especially think could be doing more to make trials more informative?

Q8 Do you have anything else you'd like to add?

If participant is a funder representative/sponsor representative/ethics committee member or other regulatory body representative:

{Use the following probe if the participant struggles or does not talk about these:

-In your opinion, at what point in the lifespan of a trial should informativeness be addressed to have the biggest impact on the number of uninformative trials (in terms of limiting the number of uninformative trials) - pre-funding, funding, conducting, reporting stages?}

Q2 Starting with a general question, what do you think makes a trial useful?

{if the respondent doesn't mention trial users (e.g., health professionals, patients) in the response, ask:

-Who are you thinking of when talking about a trial being useful? Who is the person(s) finding it useful?}

- Some US researchers created the term 'informativeness' to describe a trial that produces useful results for its users. Informativeness has five components: importance – design – integrity – feasibility – reporting. These are the things we think of when talking about the informativeness of trials.

{be ready to explain these five components if needed}

Q3 As a [type of interviewee], what sort of things does your organization do to make sure the trials it funds will be informative?

{Use the following probe if the participant struggles or does not talk about these:

-In your experience, at what point in the lifespan of a trial have issues come to your attention- pre-funding, funding, conducting, reporting stages?}

Q4 Does your organization have any Standard Operating Procedures and policies to ensure that the trials it funds are as informative as possible? Is there a standard that needs to be met?

{Use the following probes if the participant struggles or does not talk about these:

-In your description you touched on [importance/design/integrity] – could you say a bit more about this in relation to informativeness?

-In your description you didn't mention [importance/design/integrity] as conditions for informativeness – any thoughts on why not?}

Q6 And following on from that, if any issues relating to the informativeness of a trial were found, how would you address these – what would happen?

Q5 In [organization], how are issues relating to informativeness identified, how and when does your organization become aware of any issues?

© 2026 Prowse SR et al. *JAMA Network Open*.

{Use the following probe if the participant struggles or does not talk about these:

-In your experience, at what point in the lifespan of a trial have issues come to your organization's attention- pre-funding, funding, conducting, reporting stages?}

Q7 As you know, we are doing this study to try and make sure that all trials are as useful and informative as possible. In your opinion, how can the number of informative trials be increased most effectively, by whom and why?

Q8 Do you have anything else you'd like to add?

{Use the following probe if the participant struggles or does not talk about these:

-In your opinion, at what point in the lifespan of a trial should informativeness be addressed to have the biggest impact on the number of uninformative trials (in terms of limiting the number of uninformative trials) - pre-funding, funding, conducting, reporting stages?

-Is [funder name] taking any steps to address this issue internally? Which?}

Demographic questions for all participants

Before we finish, I would like to ask you some demographic questions so we can describe participants in the study, is that OK?

-{If not known} - What is your job title?

-In which country do you live in?

-What is your age?

-What is your gender?

-To make sure we get opinions and perspectives from across the whole community, we'd like to collect information on your ethnicity. With that in mind, and if you are willing, could you describe your ethnicity in your own words?

Thank you very much for your time today. Have a good day/evening/weekend!

© 2026 Prowse SR et al. *JAMA Network Open*.

Reference for interviewer

Table extract from Zarin et al 2019 – our focus is on importance, design and integrity. Table included here for interviewer to refer to if needed.

**Conditions for Informativeness**	**Red flags**	**Sample evidence indicating deficiencies**
Importance: trial hypothesis is likely to inform an important scientific, medical, or policy decision	Trial hypothesis does not have sufficient supporting evidence	Cases and systematic review of protocols suggesting insufficient preclinical evidence supporting early phase trials.
	Specific hypothesis, as reflected in trial design, is not of clinical or scientific interest	Systematic reviews suggesting many drug trials use comparators that are no longer the standard of care, or are otherwise not clinically relevant, rendering the study uninformative.
	Trial hypothesis is already resolved by previous studies, or will likely be resolved by contemporaneous trials	Cumulative meta-analyses showing that RCTs continue to be conducted long after certainty has been established about an effect.
	Resolution of trial hypothesis is unlikely to inform (or contribute to) an important medical, public health, or policy decision	Systematic reviews suggesting small, positive trials testing approved drugs are often not followed up with rigorous randomized trials.
Design: trial methods are likely to provide meaningful evidence related to study hypothesis	Trial is not adequately powered	Reviews showing a high prevalence of underpowered trials in different medical areas
	Trial design contains major unaddressed threats to internal validity	Systematic reviews suggesting that many trials have flaws in randomization, concealment of allocation, or masking
Integrity: trial is conducted and analyzed in a scientifically valid manner that is faithful to design	Study design is pre-specified in a trial protocol, but trial is not conducted, analyzed or reported in manner consistent with protocol	Systematic reviews showing that methods in protocols, registry records, publications often are inconsistent
	Study registration is not complete, specific and prior to study initiation	Evidence that close to one third of clinical trials are registered more than three months after study initiation
Feasibility: the trial is likely to be feasible		
Reporting: systems are in place to ensure timely, complete and accurate reporting		

© 2026 Prowse SR et al. *JAMA Network Open*.
